# Early selection for drought tolerance in popcorn based on gene effects estimated in seedlings

**DOI:** 10.3389/fpls.2023.1203972

**Published:** 2023-07-03

**Authors:** Carolina Macedo Carvalho, Shahid Khan, Antônio Teixeira do Amaral Junior, Valter Jário de Lima, José Gabriel de Souza Silva, Lara Moreira Catarino Fuly, Jhean Torres Leite, Divino Rosa dos Santos Junior, Flávia Nicácio Viana, Rosenilda de Souza, Henrique Duarte Vieira, Samuel Henrique Kamphorst

**Affiliations:** ^1^ Laboratório de Melhoramento Genético Vegetal, Centro de Ciências e Tecnologias Agropecuárias (CCTA), Universidade Estadual do Norte Fluminense Darcy Ribeiro - UENF, Campos dos Goytacazes, Brazil; ^2^ Crop Science Department, Crops Environment and Land Use Programme, Carlow, Ireland; ^3^ Laboratório de Fitotecnia, Centro de Ciências e Tecnologias Agropecuárias (CCTA), Universidade Estadual do Norte Fluminense Darcy Ribeiro - UENF, Campos dos Goytacazes, Brazil

**Keywords:** dominance effects, hybrids, water stress, drought, *Zea mays* L. Everta

## Abstract

Low rainfall rates are becoming increasingly frequent because of climate change, causing droughts and threatening world food security. For popcorn, drought is the most limiting abiotic factor for plant’s growth and development. Thus, the water deficit directly impacts for crop productivity. Based on knowledge of the genetic basis of traits involved in stages of popcorn germination and seedling development under water stress, genotypes with potential for adaptation to adverse growing conditions can be selected early. Therefore, data on genetic effects and combining ability of 10 popcorn parents were compiled to propose breeding strategies for the development of cultivars with greater adaptation to water stress in the early stages. Forty-five diallel hybrids were evaluated under two different water regimes, that is, water stress and full irrigation. This corresponded to a water retention capacity of 25% and 70% of the germination paper. The plants were watered daily as needed for seven days. A range of factors were evaluated, that is, germination traits including the germination speed index and germination on the seventh day; shoot traits including length and dry weight; and root system including length, dry weight, root-to-shoot ratio, maximum root number, root network area, specific and root network length, and root volume. Breeding for drought adaption in the early stages of popcorn development can be successful when hybrids are used, because of the genetic effects of dominance (ϕ_s_). These control the traits evaluated at the seedling stage. The combinations L61 x P2 and L71 x P3 were recommended, in view of the more successful performance estimated for traits related to the shoot and root system.

## 1 Introduction

Drought has become an increasingly frequent environmental stress because of climate change (Awange et al., 2016; Van Loon et al., 2016; Jehanzaib and Kim, 2020; Leite et al., 2021; Hoylman et al., 2022; Leite et al., 2022). It decreases agricultural output, resulting in lower yields in regions affected by the drought (Fahad et al., 2017; Jehanzaib and Kim, 2020; Sharif et al., 2022). Given the losses resulting from low water availability during plant development, plant breeding has become a promising alternative, for which understanding the associated morphological and physiological plant responses is crucial (Araus, 2002; Kamphorst et al., 2022). This information is required for the selection of genotypes potentially suitable under water stress (Araus et al., 2010; Adebayo et al., 2014; Altieri and Nicholls, 2017; Lima et al., 2019; De Lima et al., 2021a; De Lima et al., 2021b) to mitigate the effects of irregular rainfall (Cunha et al., 2019; Malhi et al., 2021).

For maize and other cereal species, the impacts of water stress on the development, growth, and final grain yield are not only determined by the variety, but also by the intensity of water stress (Badr et al., 2020; Kamphorst et al., 2021a; De Lima et al., 2021b; Sheoran et al., 2022; Viana et al., 2022). In the phases of seed germination and seedling growth, plant-available water level was found to be highly influential (De Carvalho and Nakagawa, 2000). Imbibition is fundamental in the germination process, given that it allows the resumption of metabolic activity in the seed and contributes to reserve mobilization, assimilation, and subsequent growth (Marcos Filho, 2015). Under water stress, germination is reduced because of the decrease in activity of hydrolytic enzymes involved in this process, such as α-amylase, β-amylase, and α-glucosidase, which are related to carbohydrate metabolism (Marcos Filho, 2015; Akinwale et al., 2017; De Abreu et al., 2019).

In Brazil, maize is predominantly cultivated in the second growing season from February to July, after the soybean harvest has occurred (Dias et al., 2018). This period coincides with the dry season, when extreme variations in rainfall can occur (Awange et al., 2016). The same is true for popcorn which has formed the focus of the present study. This specialty corn is a widely valued and consumed food, particularly in the context of leisure and cinema (Do Amaral et al., 2013; Kamphorst et al., 2021a). However, a major market could still be created in Brazil, given that the area planted has grown by 223% in the last five years (Kist et al., 2019; Kamphorst et al., 2020b; Kamphorst et al. 2020a; Kamphorst et al., 2021b; De Santos et al., 2021).

To date, the effects of water stress on trait expression in the early stages of popcorn development have not yet been studied. Therefore, understanding the genetic basis of trait performance in the seed germination and seedling development phases is important for planning breeding programs targeting the early selection of genotypes adapted to water stress (Ju et al., 2018; Dwivedi et al., 2021; Mota et al., 2021; Reis et al., 2022). Diallel crosses provide genetic information in estimates and partitioning of the combining ability of the parents (Mota et al., 2021). These data make it possible to estimate the existence and relative importance of additive, non-additive, and environmental effects on traits. Understanding these genetic mechanisms opens opportunities to increase the yield potential and optimize adaptation to water stress (Kamphorst et al., 2021a).

The present study addressed the combined ability of 10 popcorn parents which have been pre-selected for agronomic efficiency in water use, and their hybrids, produced according to a diallel mating scheme, under two contrasting water regimes, that is, water stress (WS) and well-watered (WW) conditions. This can then highlight potentially useful traits for early selection for adaptation to water stress, and identify genotypes for strategic breeding for superior segregants with drought tolerance.

## 2 Materials and methods

### 2.1 Plant material

The plant material consisted of 45 F_1_ hybrids developed using a diallel mating scheme between 10 S_7_ popcorn lines. The lines were selected from a set of 20 lines that had previously been evaluated in the field under WS. Based on the performance of the 10 lines, four were classified as agronomically efficient in terms of their water use (P2, P3, P6, and P7), with four classified as inefficient (L61, L63, L65, and L75) and two classified as intermediate (L71 and L76) (Kamphorst et al., 2018).

The genealogy of popcorn inbred lines (S7) was derived from germplasm adapted to tropical (L61, L63, L65, and L71 from the BRS-Angela population) and temperate/tropical conditions (P7, from the commercial hybrid Zélia; P2 and P3, from the compound CMS-42; P6, from the commercial hybrid IAC-112; and L54, L55, L75, and L76, from the Barão de Viçosa population) (Vittorazzi et al., 2018).

To generate the hybrids, the lines were crossed in pairs, in 6.00 m rows spaced 1.00 m apart, with the plants spaced 0.40 m apart. The ears in each line were covered with plastic bags prior to stigma exertion. The mature tassels were covered with a brown paper bag on the day preceding the crosses to avoid contamination of the crosses with undesired pollen. Approximately 65 d after planting, the plants were hand pollinated. The hybrid seeds were harvested in April 2019, at the Experimental Station of Colégio Estadual Agrícola Antônio Sarlo, in Campos dos Goytacazes, Rio de Janeiro State, Brazil (lat. 21° 42”48” S; long. 41° 20” 38” W, 14 m asl).

### 2.2 Water regimes and experimental design

**Figure 1:**
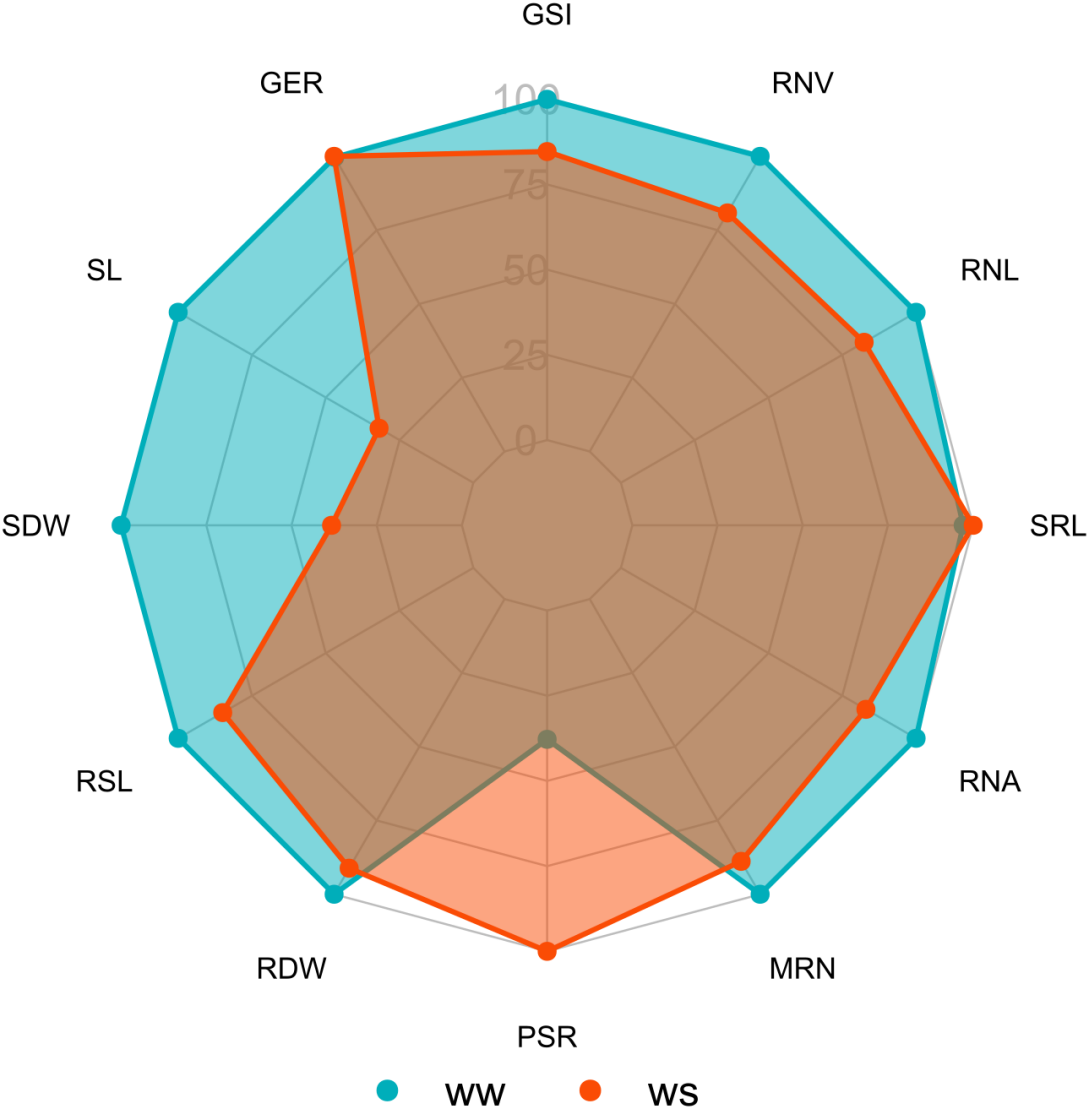
Comparison of means of traits of popcorn genotypes evaluated under water stress (WS) and well-watered (WW) conditions for germination, shoot, and root system. WS, water stress; WW, well-watered conditions; GSI, germination speed index (seed day^-1^); GER, germination on the seventh day (%); SL, shoot length (cm seedling^-1^); SDW, shoot dry weight (mg plant^-1^); RSL, root system length (cm seedling^-1^); RDW, root dry weight (mg plant^-1^); RSR, root-to-shoot ratio; MRN, maximum root number; RNA, root network area (cm^2^); SRL, specific root length (cm/cm^3^); RNL, root network length (cm); and RNV, root network volume (cm^3^).

To evaluate the early hybrid development under each water regime, two experiments were conducted in a complete randomized block design with four replications of 20 seeds per replication. The experiments were conducted at the Crop Science Laboratory of the State University of Northern Rio de Janeiro Darcy Ribeiro (LFIT/UENF). The seeds from the 45 hybrids were germinated under two water regimes (WC). These were: i) adequate substrate moisture (well-watered: WW), where the substrate (germitest paper) was wettened to a moisture content of 70% of the water retention capacity; and ii) water stress (WS), where the substrate was wettened to a moisture content of 25% of the water retention capacity. To adjust the moisture content of each WC, the dry paper was weighed and multiplied by three, which was equivalent to the weight of the water-saturated paper. Thereafter, the moisture was adjusted to 70% and 25% of the substrate retention capacity (**Supplementery Figure 1**).

Once the water content of the germitest paper had been adjusted, the seeds were arranged in four rows of five seeds, that is, 20 seeds per hybrid and replication. Each germitest paper was rolled up into a cylindrical tube and packed in two plastic bags with openings in opposite directions. This prevented water from escaping into the external environment. The paper rolls were placed in germinators, with alternating temperatures (20–30 °C) and an 8:16 h light–dark photoperiod (MAPA-Brasil, 2009). Based on the initial weight of each replication, which comprised the weight of water, seeds, and plastic cover bags, water was replenished daily to maintain the desired moisture level for each WC.

### 2.3 Traits evaluated

The traits evaluated were divided into three groups related to germination and the shoot and root systems. In each group of germination traits, germination on the seventh day (GER) and the germination speed index (GSI) were evaluated. Germination on the seventh day was evaluated as the percentage (%) of normal seedlings that emerged on the seventh day. On that day, the number of abnormal seedlings and non-germinated seeds were also counted. Seeds with no sign of germination were considered dead. Those with malformed structures and aspects of abnormalities were considered anomalous. Seedlings with strong structure formation, without any deformities and abnormalities, were recorded as normal (MAPA-Brasil, 2009). The GSI was based on the count of the number of seedlings that emerged daily until the stand stabilized. This occurred on the seventh day after sowing, with radicle emergence being considered germination. The GSI (seedlings day^−1^) was computed using the equation proposed by Maguire (1962), by counting the number of seeds with a radicle length of 0.50 cm every two days.

The shoot traits of the seedlings evaluated were the shoot length (SL) and the shoot dry weight (SDW). The shoot length (SL) (in cm seedling^−1^) was measured with a millimeter ruler, considering the distance from the seedling mesocotyl to the tip of the primary leaves (Da Silva et al., 2016). Shoot dry weight (SDW) was measured as the mean weight of all seedlings, in milligrams per normal seedling (mg seedling^−1^). For these evaluations, 10 seedlings that had been oven-dried at 70°C for 48 h were used.

Root-related traits were evaluated on the same 10 seedlings, namely the root system length (RSL), root dry weight (RDW), and root-to-shoot ratio (RSR). The length of the root system was measured from the collar to the tip of the largest primary root (in cm plant^−1^) (Da Silva et al., 2016). The root material from all replicates was then spread out and photographed (resolution 3.088 × 3.088 pixels) in a water-filled acrylic tray. This procedure reduced root overlap. The root photographs were analyzed with GiARoots (Galkovskyi et al., 2012). The software was calibrated to 93 pixels cm^−1^ and used to estimate the maximum root number (MRN), root network area (RNA, in cm^2^), specific root length (SRL, in cm/cm^3^), root network length (RNL, in cm), and root network volume (RNV, in cm^3^).

After being photographed, the RDW was estimated by weighing the material (in mg seedling^−1^) after being oven dried at 70°C for 48 h. Based on these values, divided by SDW, the RSR estimates were calculated using the equation: $RRA=\frac{PSR}{PSA}$ .

### 2.4 Statistical analyses

Individual analysis of variance was performed for each WC according to the statistical model $Y_{ij}=\mu +g_{i}+b_{j}+\epsilon_{ij}$ , where: Y_ij_ = observed value of the i-th genotype in the j-th block; μ = general constant; g_i_ = fixed effect attributed to the i-th genotype; b_j_ = random effect of block j; and ${\text{ϵ}}_{\text{ij}}$ random error associated with observation Y_ij_. The experimental variation coefficient was calculated as: $CVe=\frac{\sqrt{RMS}}{\bar{X}},\;$ where RMS corresponds to the residual mean square and $\bar{X}$ to the mean. Then, a combined analysis of variance was performed between the WCs, according to the statistical model $Y_{ijk}=\mu +G_{i}+B/WC_{jk}+WC_{j}+GWC_{ij}+\epsilon_{ijk}$ , where $\;Y_{ijk}$ : *Y_ijk_* = observation of the i-th genotype in the j-th WC and k-th block; μ = general constant; *G_i_* = fixed effect of the i-th genotype; $B/WC_{jk}=$ effect of the k-th block within *WC_j_*; *WC_j_* = fixed effect of the j-th WC; *GWC_ij_* = fixed effect of the interaction between the i-th genotype and the j-th WC; and $\epsilon_{ijk}$ = mean experimental random error associated with observation $Y_{ijk}$

The proportional reduction (%) of each variable, based on the comparison between WCs, was calculated using the formula $100-\left[\left(Y_{WS}/Y_{WW}\right)*100\right]$ , where: *Y* = overall mean of the variable, under WS and WW conditions.

For each WC, diallel analysis was performed separately, according to Griffing’s Method IV, Model 1 (Griffing, 1956), according to the expression $Y_{ij}=\mu +g_{i}+g_{j}+s_{ij}+\epsilon_{ij}$ where: Y_ij_ = mean value of the hybrid combination (i ≠ j) or the parent (i = j); μ = overall mean; g_i_ = effects of general combining ability of the i-th parent (i, j= 1, 2,…, 10); g_j_ = effects of general combining ability of the j-th parent (i, j= 1, 2,…, 10); s_ij_ = effect of specific combining ability for crosses between the i-th and j-th parent; and $\epsilon_{ij}$ mean experimental error associated with the observation of order ij.

The quadratic components (ϕ) that express the genetic variability, in terms of general (g) and specific combining ability (s), were estimated using: ϕ_g_ = (GMS – RMS)/2p and ϕ_s_ = SMS – RMS, respectively. Here, GMS is the mean square of the general combining ability, SMS is the mean square of the specific combining ability, RMS is the residual mean square, and p is the number of parents. The effects of quadratic components were expressed as a percentage of the total sum of effects.

Variance and diallel analysis were performed using the GENES program (Cruz, 2013). The graphs were constructed using the G-Biplot Package, R Studio (R Core Team, 2018). The “which won where/what” multivariate analysis was performed based on the biplot model genotype per trait (GT), using the standardized values of all the traits evaluated (GSI, GER, SL, SDW, RSL, RDW, RSR, MRN, RNA, SRL, RNL, and RNV). To generate the GT biplot graph, the R software package GGEbiplotGUI (R Core Team, 2018) was used.

## 3 Results

### 3.1 Genetic variability under different water regimes and impact of water stress

Germination traits that differed significantly under WS were GSI and GER. Under WW, the GER trait significantly differed. The experimental coefficients of variation (CV_e_) ranged from 6.73% (GER under WW) to 38.65% (GER under WS). In the combined analysis, the germination traits differed statistically only for the genotype source of variation (G) (**Table 1**). In a comparison of the WS and WW water regimes, GSI tended to decrease by 15.34% under WS, while GER increased by 0.32% under the same condition (**Figure 1**).

**Table 1:** Summary of analysis of variance, overall means, and coefficient of experimental variation (CVe) of germination-, shoot-, and root-related traits of seedlings of 45 popcorn hybrids under water stress (WS) and full irrigation (WW).

SV	WS					WW					Combined		
	GMS (DF = 44)	RMS (DF = 88)	F test	$\bar{X}$	CVe (%)	GMS (DF = 44)	RMS (DF = 88)	F test	$\bar{X}$	CVe (%)	G	WC	G^*^WC
GSI	75.62	8.13	^**^	23.90	11.93	215.50	120.32	^ns^	28.23	38.85	^**^	^ns^	^ns^
GER	629.76	38.73	^**^	90.12	6.90	776.65	36.54	^**^	89.84	6.73	^**^	^ns^	^ns^
SL	1.96	0.89	^ns^	2.73	34.51	8.68	6.87	^ns^	8.55	30.65	^**^	^**^	^ns^
SDW	0.002	0.001	^ns^	0.07	54.21	0.01	0.0005	^**^	0.18	12.19	^**^	^**^	^**^
RSL	8.79	4.56	^ns^	14.71	14.51	18.36	7.35	^*^	17.32	15.66	^**^	^**^	^**^
RDW	0.005	0.001	^**^	0.13	21.94	0.01	0.001	^**^	0.14	17.52	^**^	^ns^	^ns^
RSR	1.57	0.62	^*^	2.08	37.86	0.12	0.02	^**^	0.78	19.37	^**^	^**^	^**^
MRN	101.56	46.93	^ns^	34.22	20.02	146.37	40.13	^**^	38.51	16.45	^**^	^ns^	^ns^
RNA	389.33	196.05	^ns^	61.29	22.85	1053.46	184.15	^**^	73.84	18.38	^**^	^*^	^*^
SRL	1969.86	610.79	^**^	205.57	12.02	1769.39	450.42	^**^	199.56	10.64	^**^	^ns^	^ns^
RNL	14.24	6.04	^*^	10.75	22.86	29.45	6.54	^**^	13.04	19.60	^**^	^*^	^ns^
RNV	5.43	1.59	^**^	5.48	23.02	10.22	2.09	^**^	6.78	21.29	^**^	^*^	^ns^

SV, source of variation; WS, water stress condition; WW, well-watered condition; X, mean; GMS, genotype mean square; RMS, residual mean square; CVe (%), coefficient of experimental variation; G, genotype; WC, water regime; G*WC, water*condition interaction; GSI, germination speed index (seed day^-1^); GER, germination on the seventh day (%); SL, shoot length (cm seedling^-1^); SDW, shoot dry weight (mg plant^-1^); RSL, root system length (cm seedling^-1^); RDW, root dry weight (mg plant^-1^); RSR, root-to-shoot ratio; MRN, maximum root number; RNA, root network area (cm^2^); SRL, specific root length (cm/cm^3^); RNL, root network length (cm); and RNV, root network volume (cm^3^). Mean square effect represented by ns, *, and **, when not significant, significant at p< 0.05, and significant at p< 0.01, respectively, by the F Test.

Of the shoot traits, only SDW under WW differed significantly (**Table 2**). Regarding CVs, there was a variation from 54.21% (SDW under WS) to 12.19% (SDW under WW) (**Table 1**). The combined analysis showed significant differences for the source of variation G and water condition (WC) for the traits SL and SDW and for the genotype × water regime interaction (G*WC) for SDW (**Table 1**). Comparing the water conditions (WCs), under WS, PSA was reduced by 61.75% and CPA by 68.06%, in relation to the WW condition (**Figure 1**).

**Table 2:** Summary of diallel analysis for germination-, shoot-, and root-related traits in popcorn hybrid seedlings evaluated under water stress (WS) and well-watered (WW) conditions.

SV	WS					WW				
	GCA		SCA		Residue	GCA		SCA		Residue
	MS	ϕ_g_	MS	ϕ_s_	-	MS	ϕ_g_	MS	ϕ_s_	-
GSI	^**^	4.96	^**^	18.09	8.13	^ns^	3.13	^ns^	33.44	120.32
GER	^**^	32.65	^**^	180.51	38.73	^**^	37.93	^**^	232.10	36.54
SL	^ns^	0.05	^ns^	0.34	0.89	^ns^	0.30	^ns^	0.14	6.87
SDW	^ns^	0.00003	^ns^	0.0002	0.001	^**^	0.0005	^**^	0.001	0.0005
RSL	^*^	0.52	^ns^	0.70	4.56	^**^	1.80	^ns^	0.91	7.35
RDW	^**^	0.001	^**^	0.001	0.001	^**^	0.001	^**^	0.001	0.001
RSR	^**^	0.10	^ns^	0.19	0.62	^**^	0.02	^ns^	0.01	0.02
MRN	^ns^	4.76	^ns^	13.10	46.93	^**^	11.63	^*^	20.61	40.13
RNA	^**^	38.98	^ns^	0.82	196.05	^**^	101.98	^**^	154.49	184.15
SRL	^**^	107.27	^*^	348.85	610.79	^**^	95.82	^**^	355.60	450.42
RNL	^**^	0.92	^ns^	1.54	6.04	^**^	2.68	^**^	4.09	6.54
RNV	^**^	0.46	^ns^	0.67	1.59	^**^	0.96	^**^	1.43	2.09

SV, source of variation; WS, water stressed; WW, well-watered; MS, mean square; GSI, germination speed index; GER, germination on the seventh day; SL, shoot length; SDW, shoot dry weight; RSL, root system length; RDW, root dry weight; RSR, root-to-shoot ratio; MRN, maximum root number; RNA, root network area; SRL, specific root length; RNL, root network length; RNV, root network volume; SCA, specific combining ability; and GCA, general combining ability. Mean square effect represented by ns, *, and **, when not significant, significant at 5%, and at 1% probability, respectively, by the F Test.

The root-related traits with significant differences were RDW, SRL, and RNV under both WCs and RSR, MRN, RNA, and RNL under WW conditions only. RSL and RSR differed significantly for the three sources of variation (G, WC, and G*WC) and the traits RDW, MRN, RNA, and SRL only for the source of variation G. Meanwhile, RNL and RNV showed significance for sources of variation G and WC. Comparing WS to the WW condition, there was a decrease in CRS of 15.09%, in RNA of 16.99%, in RNL of 17.60%, and in RNV of 19.16% (**Figure 1**). However, RNA increased by 164.54% (**Figure 1**).

### 3.2 Significance of estimates of general (GCA) and specific (SCA) combining ability and percentage importance of quadratic components (ϕ) under different water regimes

**Figure 2:**
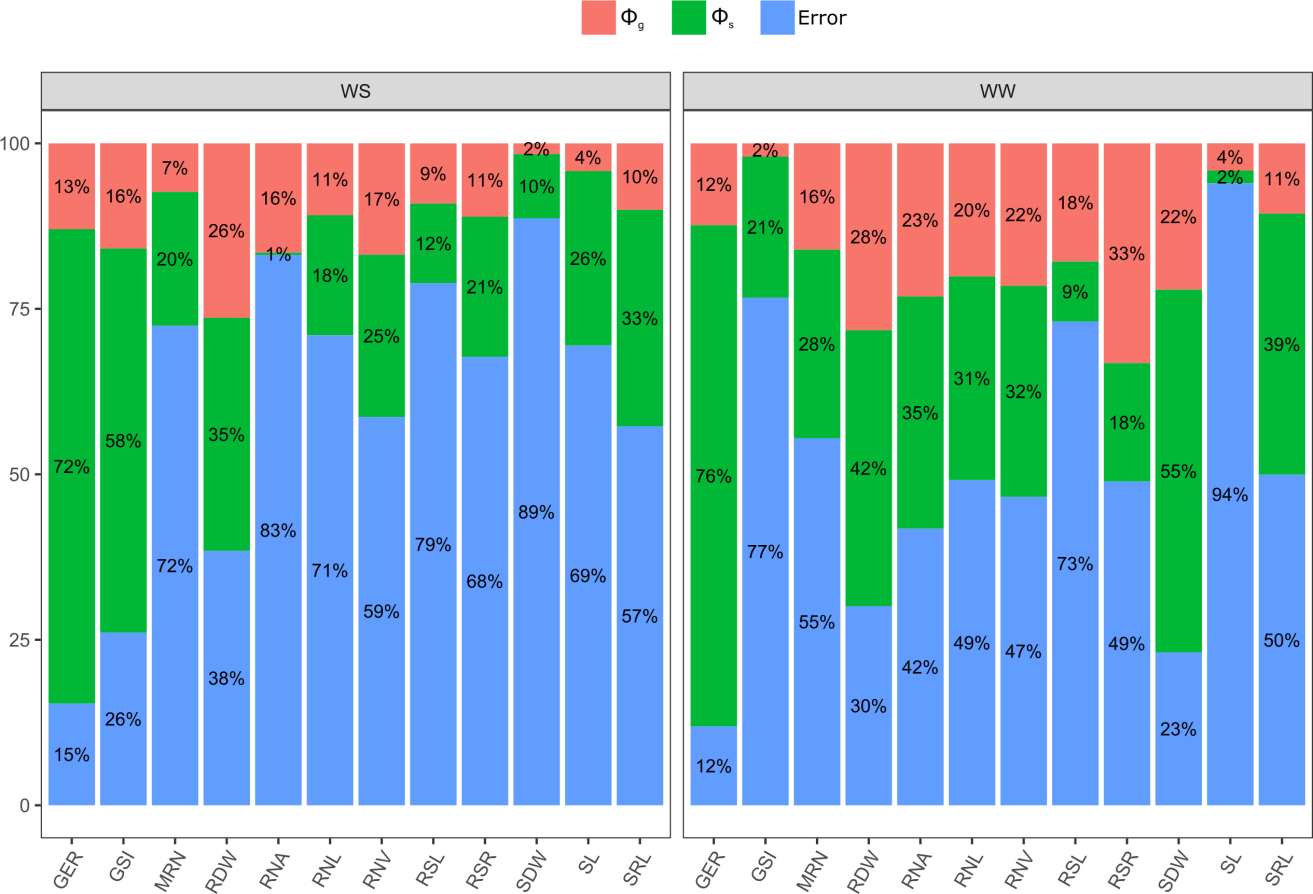
Relevance of quadratic components for germination-, shoot-, and root-related traits of popcorn genotype seedlings evaluated under water stress (WS) and well-watered (WW) conditions. GSI, germination speed index; GER, germination on the seventh day; SL, shoot length; SDW, shoot dry weight; RSL, root system length; RDW, root dry weight; RSR, root-to-shoot ratio; MRN, maximum root number; RNA, root network area; SRL, specific root length; RNL, root network length; and RNV, root network volume.

Germination traits with significant differences for GCA estimates were GSI under WS and GER under WW conditions (**Table 2**). Significance was observed for SCA in GSI and GER under WS and GER under WW conditions (**Table 2**). It was found that the quadratic components associated with SCA (ϕ_s_) were the most important in explaining the observed variability in GSI under WS and in GER under WW conditions (**Figure 2**). Given the high percentages of residues, a strong environmental influence was detected for GSI, with a magnitude of 77% (**Table 2**).

For the shoot trait SDW under WW, significant differences for GCA estimates and significance for SCA were observed (**Table 2**). The quadratic components associated with SCA (ϕ_s_) explained most of the variability observed in SL under WS (**Figure 2**). As a result of the high residual percentages, a strong environmental influence on SL and SDW was observed with estimates of 94% and 89%, respectively (**Table 2**).

The root-related traits that differed significantly in GCA estimates were RDW, RSR, RNA, SRL, RNL, and RNV under both WCs (**Table 2**). Under both WCs, significant SCA effects were observed for RDW and RNA, SRL, RNL, and RNV under WW conditions (**Table 2**). The quadratic components associated with SCA (ϕ_s_) explained most of the observed variability in RDW, MRN, and SRL under both WCs (**Figure 2**). As a result of the strong estimates of experimental residues, consistent environmental influences on CRS and RNA were detected, as shown by a CV_e_ of 79% and 83%, respectively (**Table 2**).

### 3.3 General combining ability of the genotypes evaluated under different water regimes

**Figure 3:**
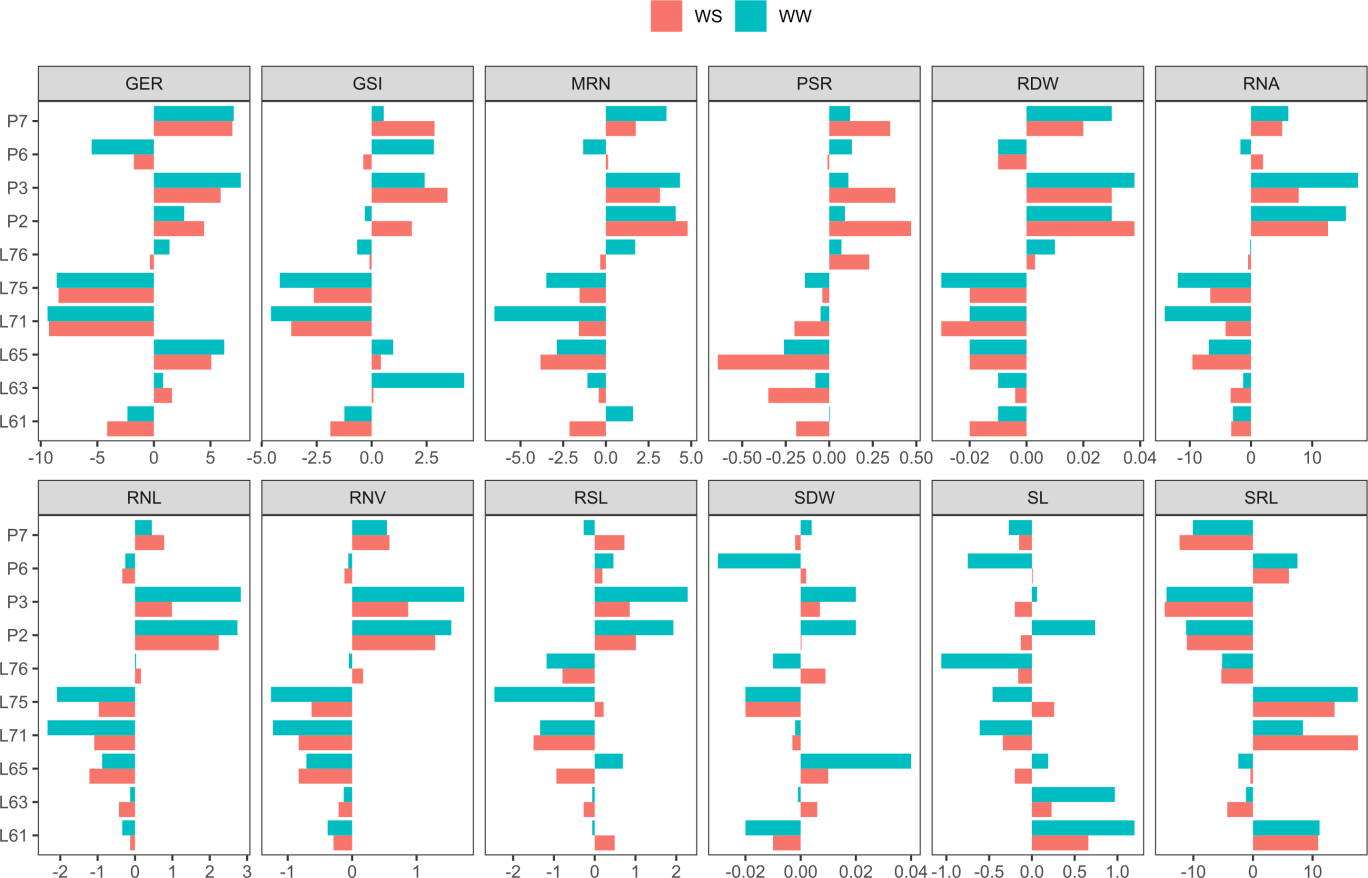
Estimates of general combining ability (GCA) for germination-, shoot-, and root-related traits, evaluated in popcorn seedlings under water stress (WS) and well-watered (WW) conditions. GSI, germination speed index (seed day^-1^); GER, germination on the seventh day (%); SL, shoot length (cm seedling^-1^); SDW, shoot dry weight (mg plant^-1^); RSL, root system length (cm seedling^-1^); RDW, root dry weight (mg plant^-1^); RSR, root-to-shoot ratio; MRN, maximum root number; RNA, root network area (cm^2^); SRL, specific root length (cm/cm^3^); RNL, root network length (cm); and RNV, root network volume (cm^3^).

For GSI, under both WCs, the parents with positive GCA estimates were L63 (0.08 under WS and 4.21 under WW), L65 (0.42 under WS and 0.98 under WW), P3 (3.45 under WS and 2.41 under WW), and P7 (2.86 under WS and 0.55 under WW). For GER, the parents L63 (1.60 under WS and 0.81 under WW), L65 (5.07 under WS and 6.22 under WW), P2 (4.44 under WS and 2.68 under WW), P3 (5.90 under WS and 7.68 under WW), and P7 (6.94 under WS and 7.06 under WW) had positive GCA effects (**Figure 3**).

For shoot-related traits, the parents with positive GCA estimates under both WCs were L61 (0.66 under WS and 1.29 under WW) and L63 (0.23 under WS and 0.97 under WW) for SL. For SDW, estimates were positive for L65 (0.01 under WS and 0.04 under WW), P2 (0.0003 under WS and 0.0183 under WW), and P3 (0.0073 under WS and 0.0233 under WW) (**Figure 3**).

Regarding the root-related traits, the parents with positive GCA effects under both WCs were P2, P3, and P6 for CRS; L76, P2, P3, and P7 for RDW; and L76, P2, P3, and P7 for RSR. For MRN, positive estimates were predicted for P2, P3, and P7. For RNA, the same situation was detected for P2, P3, and P7. In SRL, positive GCA values were found for L61, L71, L75, and P6. For RNL, the parents L76, P2, P3, and P7 had positive GCA estimates and, equally, P2, P3, and P7 in relation to RNV (**Figure 3**).

### 3.4 Estimates of specific combining ability and hybrid means under different water regimes

**Figure 4:**
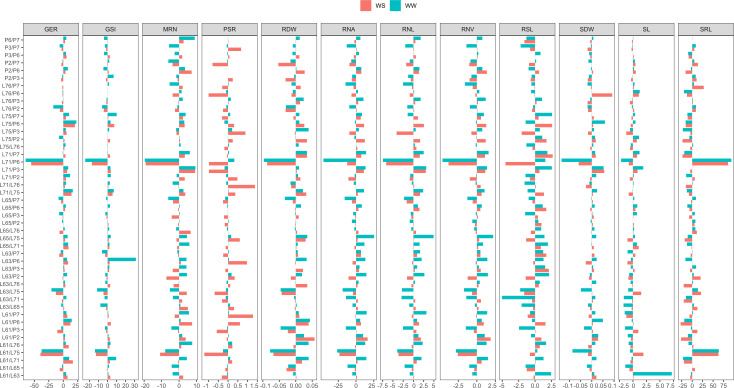
Estimates of specific combining ability (SCA) of germination-, shoot-, and root-related traits of seedlings of 45 popcorn hybrids evaluated under water stress (WS) and well-watered (WW) conditions. GSI, germination speed index (seed day^-1^); GER, germination on the seventh day (%); SL, shoot length (cm seedling^-1^); SDW, shoot dry weight (mg plant^-1^); RSL, root system length (cm seedling^-1^); RDW, root dry weight (mg plant^-1^); RSR, root-to-shoot ratio; MRN, maximum root number; RNA, root network area (cm^2^); SRL, specific root length (cm/cm^3^); RNL, root network length (cm); and RNV, root network volume (cm^3^).

**Figure 5:**
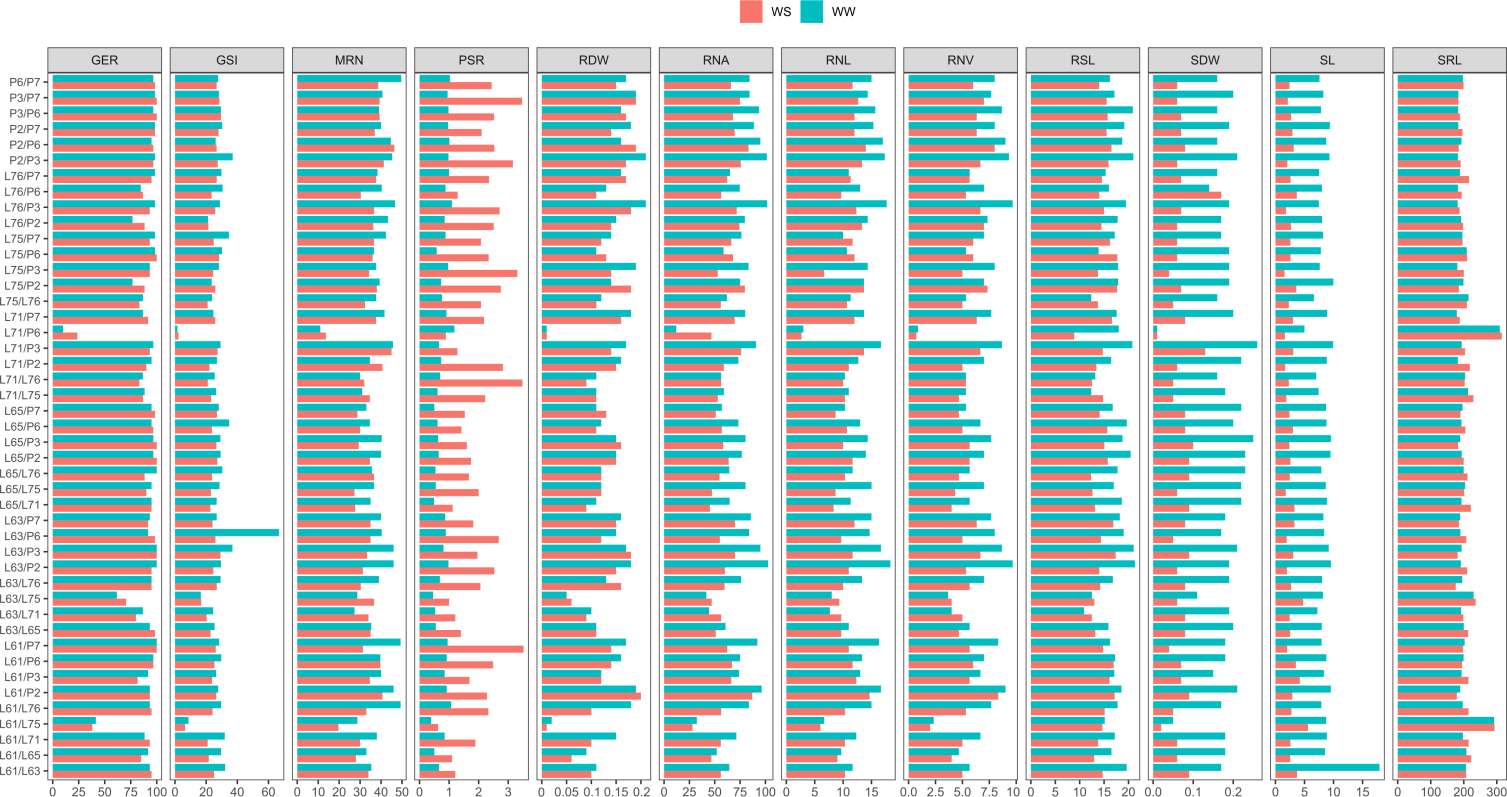
Mean estimates of germination-, shoot-, and root-related traits of seedlings of 45 popcorn hybrids evaluated under water stress (WS) and well-watered (WW) conditions. GSI, germination speed index (seed day^-1^); GER, germination on the seventh day (%); SL, shoot length (cm seedling^-1^); SDW, shoot dry weight (mg plant^-1^); RSL, root system length (cm seedling^-1^); RDW, root dry weight (mg plant^-1^); RSR, root-to-shoot ratio; MRN, maximum root number; RNA, root network area (cm^2^); SRL, specific root length (cm/cm^3^); RNL, root network length (cm); and RNV, root network volume (cm^3^).

For germination traits, under both WCs, the hybrids with simultaneously highest SCA estimates and means (among the 15 highest values), were L75 × P6 and L63 × P3 for GSI and L61 × P6, L61 × P7, and L75 × P6 for GER (**Figures 4**, **5**).

For shoot traits and considering both WCs together, the hybrids with more marked SCA estimates and means among the 15 highest values were L65 × L71 and L71 × P3 for SL and L61 × P2, L71 × P3, and L71 × P7 for SDW (**Figures 4**, **5**).

When evaluating the root traits together, concomitantly under both WCs, the hybrids with the highest SCA and mean values among the 15 highest values were L63 × P3 and L65 × P6 for CRS; L61 × P2, L71 × P7, and L76 × P3 for RDW; L61 × P7, L63 × P2, and L75 × P3 for RSR; L71 × P3, L71 × P7, L75 × P7, P2 × P6, and P6 × P7 for MRN; L61 × P2, L71 × P3, L76 × P3, and P2 × P6 for RNA; L61 × L75, L63 × L65, L63 × L75, L65 × L76, and L71 × P6 for SRL; L61 × P2, L63 × P7, L71 × P3, and P2 × P6 for RNL; and L61 × P2, L71 × P3, L71 × P7, and P2 × P6 for RNV (**Figures 4**, **5**).

### 3.5 Multivariate analysis - GT biplot

**Figure 6:**
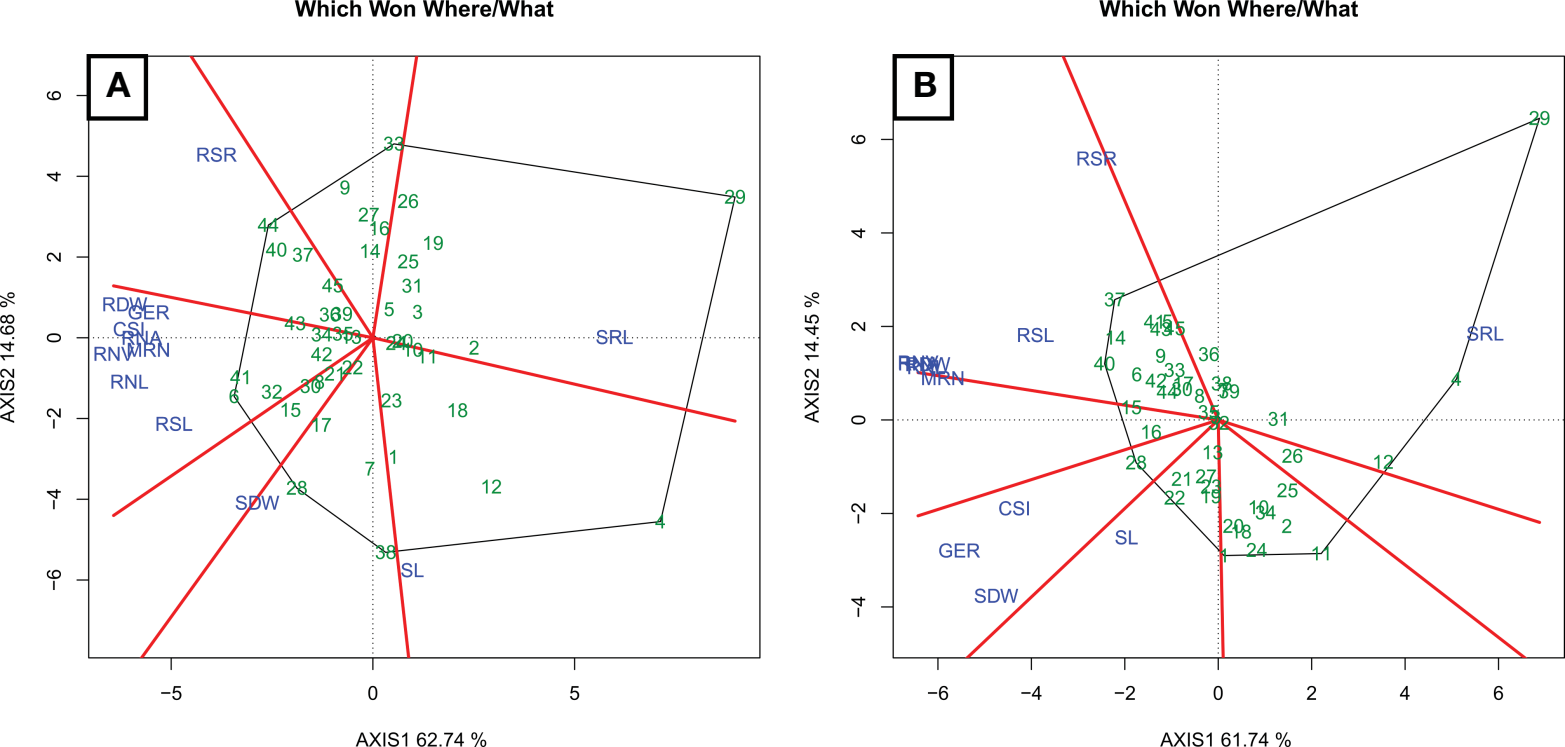
Biplot “which won where/what” diagram of water stress **(A)** (WS) and well-watered **(B)** (WW) conditions. GSI, germination speed index; GER, germination on the seventh day; SL, shoot length; SDW, shoot dry weight; RSL, root system length; RDW, root dry weight; RSR, root-to-shoot ratio; MRN, maximum root number; RNA, root network area; SRL, specific root length; RNL, root network length; and RNV, root network volume.

The main principal components had values exceeding 70%, regardless of the WC (**Figure 6**). Under WS (**Figure 6A**), the sum of the values of the first two principal components was 77.42% and 76.19% under WW (**Figure 6B**).

Under WS, five groups were formed (**Figure 6A**). The first only included RSR, for which the estimate of hybrid P3 × P7 (44) stood out. The second group, with high estimates for hybrid L71 × P7 (30), consisted of trait SRL. In the third group, with trait SL, the estimate of hybrid L61 × L75 (4) was the most appropriate. In the fourth group, with SDW, the estimates of hybrids L71 × P3 (28) and L76 × P6 (38) were the highest. In the fifth group, which comprised GER, GSI, RDW, RNA, RNV, MRN, RNL, and RSL, the hybrid estimates were highest for L61 × P2 (6) and P2 × P6 (41).

Under WW conditions, five groups formed (**Figure 6B**). The first comprised RSL, RSR, RDW, RNA, RNV, and RNL, with appropriate estimates for the hybrids L76 × P3 (37) and P2 × P3 (40). In the second group, with trait SRL, the hybrids L61 × L65 (4), L76 × L75 (12), and L71 × P6 (29) were noteworthy. In the third group, for SL, the estimates of hybrid L65 × P3 (22) stood out. In the fourth group, which included SDW, GER, and GSI, hybrid L71 × P3 (28) performed most successfully. In the fifth group, with MRN, no high estimates were predicted for any hybrid.

## 4 Discussion

### 4.1 Genetic variability under different water regimes and impact of water stress

For the germination traits GSI and GER, the applied water restriction was insufficient to differentiate WS from the WW condition, although GSI tended to decrease. However, GER was not influenced. In a similar study on maize seedlings, De Abreu et al. (2019) observed no significant differences between water stress and well-irrigated conditions for the same traits. The trend towards a decrease in GSI was because of a greater mobilization of the metabolism to mitigate the adverse effects of drought. This led to a greater energy expenditure and favored adaptation to WS during seed germination if the process occurred under WS (Vaz-de-Melo et al., 2012). In relation to GER, estimates are expected to decrease, because, given factors induced by water limitation, fewer reserves are mobilized, with less synthesis and enzymatic activity, with consequent changes in cell turgor (Bewley and Black, 1994).

For the seedling shoot traits, SDW and SL, the water restriction applied was sufficient to differentiate WS from WW conditions by causing an impact of a more than 60% reduction in these traits. The response in the shoot traits of the seedlings was one of the main causes of low productivity, for directly causing photosynthetic rate decline (Lisar et al., 2012). The observed decrease in SDW and SL was consistent with the earlier description as a response to water stress that led to a decrease in vegetative growth and leaf area, as well as leaf curling and reduced turgor (Pezzopane et al., 2015).

The root traits RSL, RNA, RNL, and RNV were affected by the experimentally imposed water stress, as shown by the significant difference in the source of variation for WC. The root system is related to the plant perception of water deficit in the soil, causing physiological changes in different metabolic pathways (Rahnama et al., 2011). According to Sharp et al. (2004), the lower values of root-related traits were due to root thinning. Thinning is a response that has already been observed in plants exposed to WS and is a morphological modification considered an adaptive response as a result of restriction of the lateral expansion rate of cortex cells (Sharp et al., 2004). However, the increase in RSR indicated a response of the root system to artificial water stress. The plant tends to decrease investments in leaf growth and intensify root development (Ribeiro, 2021). According to Lisar et al. (2012), under WS, the RSR increases because of root elongation, which deepens the root system to take up water and, consequently, to maintain the osmotic pressure. Root elongation was also observed by De Abreu (2016) in an evaluation of the same trait under water stress.

### 4.2 Implications for breeding in view of the percentage importance of quadratic components (ϕ) under different water regimes

Under water stress, the mode of action of agronomically important and other secondary traits is fundamental for the elaboration of more efficient breeding strategies (Derera et al., 2008). Knowledge about the genetic mode of action is key to successful selection (Hallauer et al., 2010). Germination-, shoot-, and root-related traits of maize seedlings normally have high coefficients of variation, as those observed by De Abreu (2016). The high experimental coefficients influenced the importance of quadratic components, in many cases driving up the estimated residual percentage to higher values than _s_ and _g_. In general, this finding was repeated for traits evaluated in this study, aside from GER and GSI under WS; and for GER, SDW, and RDW under WW. When comparing only the percentage importance of the quadratic components associated with general (_g_) and specific (_s_) combining ability, the effects of _s_ explained most of the genetic variability. In these cases, the use of hybrids is recommended, as the beneficial effects of allelic complementation are exploited.

Regarding the estimates of the mean square effects, there was no change between the WCs in the most important quadratic component for the traits evaluated in this study. This shows that the mode of trait expression is the same under both WCs. According to Lima et al. (2019), under WS and WW conditions, the genetic effects of grain yield inheritance and yield components in popcorn did not change between the tested water regimes. These results were corroborated by Kamphorst et al. (2021a). These studies suggest that the same breeding methods could be applied under both WCs, as, for example, are being proposed in this study. The same arguments were supported by Kamphorst et al. (2022) in an evaluation of growth, photosynthesis, transpiration, and root architecture traits of adult popcorn plants under different water regimes. These results are parallel to those reported here, since the genetic effects of dominance were also the most important in the trait expression of popcorn seedling growth.

### 4.3 Estimates of general and specific combining ability under different water regimes

The parents studied in this research had been selected for higher grain yield under drought during physiological maturation. The parents P2 and P3, previously classified as tolerant, stood out with the most successful performances for traits of germination and seedling development, based on the GCA means. Until then, it was unknown how these studied lines would perform in the early growth phase. The observed performance for grain yield, evaluated at the end of the cycle, was consistent with that observed during seed germination and seedling development. In general, the best lines preformed similarly under both tested water regimes (**Figure 3**).

Based on the general combining ability under both water regimes, for the germination traits, lines L63, L65, P3, and P7 can be recommended as parents of superior hybrids to mitigate the harmful effects of WS in popcorn. For shoot traits, there were no lines that stood out under both WCs together. For the root-related traits, considered together, lines P2, P3, and P7 are recommended as parents of superior hybrids, with a view to reducing the harmful effects of drought on early popcorn development. Overall, for all groups of traits, lines P2 and P3 are recommended as parents of superior hybrids for water stress tolerance.

From a broader perspective, the hybrids performed similarly under both the water regimes tested (**Figures 4**, **5**). From evaluation of the combined shoot and root traits, hybrids L71 × P3 and L61 × P2 are recommended. These hybrids responded most successfully to water stress, in terms of a stronger performance, both of roots and shoots, and best adaptation to WS was observed in the root-related traits.

The hybrids L61 × P2 and L71 × P3 were derived from lines P2 and P3, classified as tolerant, while line L61 was classified as susceptible and L71 as intermediate. This shows that the intolerant and intermediate parents, although undesirable per se for stronger performance in a water-stressed environment, represent hybrids that perform well for adaptation to dry environments, given the favorable allelic complementation observed. According to Cruz et al. (2012), the hybrid combination with the highest SCA estimate can be concluded to be the most suitable if it can be derived from a cross in which at least one of the parents has a high GCA. Therefore, our results confirm the usefulness of diallel hybrids for the development of more sustainable popcorn crops in water-stressed environments given that both parents of the most outstanding F_1_ lines in terms of SCA had high GCA estimates.

### 4.4 Relevance of traits to discriminate water-stress-adapted genotypes

In the early stage of popcorn development, the root–shoot ratio, as well as specific root length, shoot length, and shoot dry weight of the seedlings were important traits to differentiate the WS tolerant genotypes. Hybrid P3 × P7 (44) was noted for the high mean RSR; hybrid L71 × P7 (30) with a high mean SRL; hybrid L61 × L75 (4) with high mean SL; and the hybrids L71 × P3 (28) and L76 × P6 (38) with high SDW means. The other traits—GER, GSI, SDW, RSL, RDW, MRN, RNA, RNL, and RNV—seem to be closely related and were not adequate for genotypic discrimination. Meanwhile, under the WW condition, the traits RSL, RSR, SRL, SL, GER, and GSI were important to discriminate the studied genotypes. The hybrids L76 × P3 (37) and P2 × P3 (40) were with high RSL and RSR means; hybrid L71 × P6 (29) was with a high SRL mean; and hybrid L71 × P3 (28) was with high means of SDW, REE and GSI. The traits RSL, RSR, SRL, SL, GER and GSI seem to be closely related and they satisfactorily differentiated genotypes for WS adaptation. Therefore, they can be recommended for future studies to be tested from the perspective of being effectively used in early selection, as applied to popcorn in this study.

## 5 Conclusions

Breeding for drought adaption in the early stages of popcorn development can be successful when hybrids are used, because of the genetic effects of dominance (s), which control the traits evaluated in the seedling stage. The combinations L61 × P2 and L71 × P3 were recommended, in view of the more successful performance estimated for traits related to the shoot and root system.

For future investigations for the differentiation of popcorn genotypes in the germination and seedling emergence stages, regardless of the water regime applied, the evaluation of the traits specific to root length, root–shoot ratio, and shoot dry weight is strongly suggested.

## Data availability statement

The raw data supporting the conclusions of this article will be made available by the authors, without undue reservation.

## Author contributions

Conceptualization, SHK. Methodology, CC, SK, JdS, LF, JL, DdS, FV, RS, HV, and SHK. Investigation, CC, SK, JdS, LF, JL, DdS, FV, RS, HV, and SHK. Writing - original draft preparation, CC, and SK; funding acquisition, SK and AdA. All authors contributed to the article and approved the submitted version.

## Supplementary material

Supplementary Figure 1 Emergence of popcorn seedlings at 7 days after germination in water conditions of 25% **(A)** and 70% **(B)** of substrate moisture retention capacity, respectively.
