# Supplementary figures and images for: Early selection for drought tolerance in popcorn based on gene effects estimated in seedlings
Source: Front Plant Sci. 2023 Jul 3;14:1203972. doi: 10.3389/fpls.2023.1203972 (PMC10350647; doi:10.3389/fpls.2023.1203972)

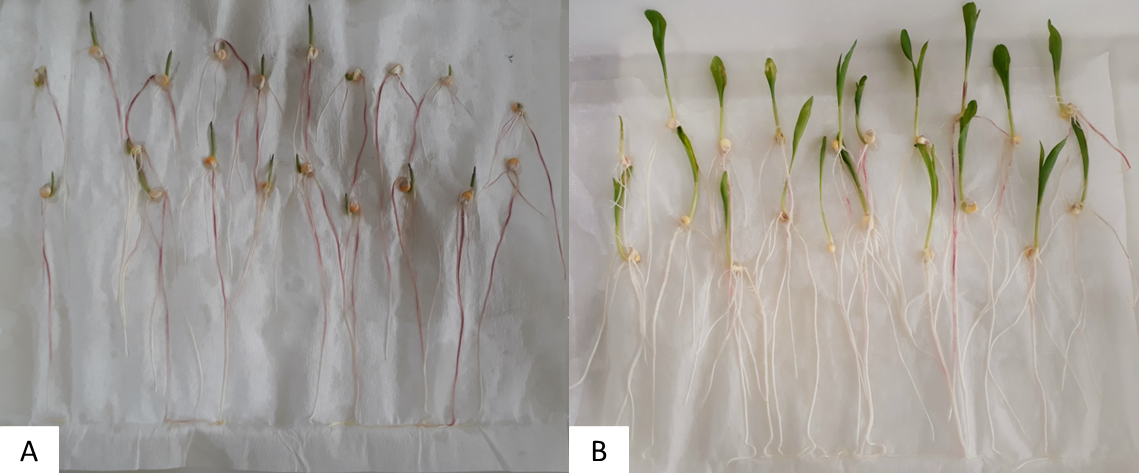

Supplement: Supplementary Figure 1 — Emergence of popcorn seedlings at 7 days after germination in water conditions of 25% (A) and 70% (B) of substrate moisture retention capacity, respectively. [file Image_1.tif]
